# Multimodal Monitoring Results Around Delayed Cerebral Infarction After Aneurysmal Subarachnoid Hemorrhage

**DOI:** 10.1007/s12975-026-01435-8

**Published:** 2026-04-25

**Authors:** Michael Veldeman, Stefan Yu Bögli, Ihsane Olakorede, Catharina Conzen-Dilger, Miriam Weiss, Nick Kastenholz, Jan Killermann, Erta Beqiri, Katharina Seyfried, Charlotte S Weyland, Hans Clusmann, Gerrit Alexander Schubert, Anke Hoellig, Peter Smielewski

**Affiliations:** 1 Department of Neurosurgery, RWTH Aachen University Hospital, Pauwelsstrasse 30, Aachen, 52074 Germany; 2 Brain Physics Laboratory, Division of Neurosurgery, Department of Clinical Neurosciences, University of Cambridge, Cambridge, UK; 3 Department of Neurology and Neurocritical Care Unit, Clinical Neuroscience Center, University Hospital and University of Zurich, Zurich, Switzerland; 4 Department of Neurosurgery, Center for Neurosurgery, Faculty of Medicine and University Hospital, University of Cologne, Cologne, Germany; 5 Department of Neurosurgery, Cantonal Hospital Aarau, Aarau, Switzerland; 6 Faculty of Medicine, University of Bern, Bern, Switzerland; 7 Department of Diagnostic and Interventional Neuroradiology, RWTH Aachen University Hospital, Aachen, Germany

**Keywords:** Aneurysmal subarachnoid hemorrhage, Brain tissue oxygen, Cerebral autoregulation, Cerebral infarction, Cerebral microdialysis, Delayed cerebral ischemia, Glutamate, Glycerol

## Abstract

Background– Delayed cerebral ischemia following aneurysmal subarachnoid hemorrhage is a major cause of secondary brain injury, with progression to infarction occurring in a subset of patients. Although multimodal neuromonitoring provides continuous physiological data, biomarkers characterizing the transition from delayed cerebral ischemia to infarction remain insufficiently defined. Methods– In this prospective single-center study, 69 patients with aneurysmal subarachnoid hemorrhage (2014–2020) underwent invasive neuromonitoring. Twenty-four patients developed delayed cerebral ischemia–related infarction, while 45 served as controls. Monitoring included brain tissue oxygenation, cerebral microdialysis, intracranial pressure, and pressure reactivity index assessment. Temporal dynamics of physiological parameters surrounding infarct detection were analyzed using generalized additive models. A Random Forest classifier evaluated multimodal biomarker patterns across a 73-hour window encompassing infarct detection. Results– Distinct metabolic disturbances preceded infarction development. Glutamate concentrations increased markedly beginning approximately 48 h before infarct detection, consistent with excitotoxic injury. Glycerol levels rose persistently after infarct detection, indicating membrane breakdown. Pressure reactivity index deteriorated after infarction, while brain tissue oxygenation increased post-detection. The Random Forest model demonstrated good discriminative performance (area under the curve 0.814), with temporal glutamate patterns contributing most to feature importance. Averaged biomarker trends outperformed instantaneous measurements. Conclusions– Multimodal neuromonitoring reveals characteristic physiological patterns associated with delayed cerebral ischemia–related infarction. Integration of metabolic, oxygenation, and cerebrovascular reactivity markers enhances characterization of infarction development, warranting validation in larger cohorts. This study was retrospectively registered with the German Clinical Trials Registry(DRKS00030505) on the third of January 2023.

## Introduction

Aneurysmal subarachnoid hemorrhage (SAH) is a life-threatening condition often complicated in the subacute phase by delayed cerebral ischemia (DCI) [1]. Although the pathophysiology of DCI is not fully elucidated, it is recognized as a multifactorial supply demand mismatch, involving macro- and microvascular disturbances, neuroinflammatory cascades, and cortical spreading depolarizations [2]. In a subset of patients, DCI results in irreversible cerebral injury in the form of progression to infarction [3].

In unconscious patients, invasive neuromonitoring, including brain tissue oxygenation (PtiO₂) and cerebral microdialysis, provides valuable physiological insight and may support the early identification of DCI [4]. While DCI diagnosis in clinically assessable patients relies on neurological examination and imaging, diagnostic criteria in comatose patients remain ill-defined. Consequently, reliable biomarkers for impending infarction are needed.

First-tier therapy for suspected DCI typically involves induced hypertension to augment regional cerebral perfusion in regions at risk [5]. Although this approach is supported primarily by observational studies, it remains a widely adopted strategy and is endorsed by current clinical guidelines [6].

The metabolic perturbations leading to infarction following cerebral ischemia have been characterized primarily in animal models of middle cerebral artery occlusion and in human studies examining blood and cerebrospinal fluid [7]. However, due to ethical and technical constraints, our knowledge of tissue metabolism during the hyperacute phase of ischemia in humans is limited. As a result, our understanding of metabolic derangement in the critical phase preceding infarction remains incomplete. In addition, pathophysiology and possible underlying cellular derangement might differ vastly between occlusive thrombo-embolic stroke and DCI progressing to DCI-related infarction.

Prior CMD studies in SAH provided important proof-of-concept but were frequently performed within earlier vasospasm-based diagnostic and treatment paradigms and often summarized measurements over broad time windows [8, 9]. Here, we leverage continuous multimodal monitoring in a contemporary care context and align physiological data to infarct detection to characterize peri-event dynamics, focusing on temporal trends (e.g., rolling averages and slopes) rather than single thresholds. This design aims to improve interpretability of neuromonitoring signals in a timeline disease in which actionable deterioration may be gradual.

Disturbances of cerebral autoregulation following SAH have also been identified as contributing factors to secondary ischemic injury and progression to infarction [10, 11]; however, these dynamics have not been systematically characterized around the time point of DCI.

Unlike thromboembolic ischemic stroke, in which monitoring, if used, is initiated after symptom onset, patients with SAH are frequently monitored with multimodal neuromonitoring prior to the development of infarction. This unique context presents a rare opportunity to capture the evolution of cellular metabolic dysfunction during the transition from DCI to DCI-related infarction.

## Objectives

We aim to characterize the metabolic and autoregulatory changes associated with the development of DCI-related infarction. Specifically, we will examine alterations in key microdialysis biomarkers, including glutamate and glycerol, in relation to dynamic changes in PtiO₂ and cerebrovascular reactivity as assessed by the pressure reactivity index (PRx) [12]. Our objective is to quantify both the magnitude and temporal evolution of these changes surrounding the detection of DCI-related infarction.

## Methods

### Patient Population

Since 2014, a prospective, single-center registry has been maintained at RWTH Aachen University Hospital, capturing all consecutive cases of SAH. Inclusion required radiological confirmation of aneurysmal origin, based on computed tomography angiography or digital subtraction angiography. Only patients with documented informed consent were enrolled. In cases where patients were unable to consent due to poor clinical condition, consent was obtained from a legally authorized representative or next of kin, in accordance with ethical guidelines.

The registry has received approval from the local ethics committee (EK 14/062 and EK 22/371) and is registered retrospectively with the German Clinical Trials Registry (DRKS00030505). Data from this cohort, either independently or in combination with datasets from collaborating institutions, have contributed to previously published studies [11, 13–17].

### Clinical Management

As per institutional protocol, aneurysms were treated within 24 h via surgical clipping or endovascular coiling, and all patients received prophylactic oral nimodipine. Acute hydrocephalus was managed with external ventricular drainage placement. In patients who were not assessable neurologically after a wake-up test or who later deteriorated neurologically, invasive neuromonitoring was initiated. High-grade SAH patients (Hunt & Hess IV and V) were equipped with neuromonitoring prior to securing the aneurysm.

Microdialysis catheters (71 High Cut-Off Brain Microdialysis Catheter, µdialysis) and combined brain tissue oxygenation/intracranial pressure probes (Neurovent PTO, Raumedic, Germany) were placed on the side of the ruptured aneurysm. For midline aneurysms, probes were inserted on the side with the most pronounced subarachnoid hemorrhage. All probes were implanted via a double-lumen bolt, approximately 1 cm lateral to Kocher’s point, targeting the frontal watershed region aiming to sample both the anterior and middle cerebral artery territories.

### Delayed Cerebral Ischemia and infarction

The evaluation of DCI was supported by multimodal neuromonitoring. Clinical triggers for further imaging included elevated transcranial Doppler velocities (> 120 cm/s) [18], occurrence of Brain tissue hypoxia defined as P_ti_O_2_ <20 mmHg, or metabolic derangement, in particular an elevated lactate-to-pyruvate ratio (LPR > 40). In such cases, CT perfusion (CTP) imaging was performed to assess for ischemic deficits. Upon confirmation, first-line treatment consisted of inducing euvolemic hypertension via norepinephrine infusion to augment cerebral perfusion. Follow-up CTP imaging was performed within 6 to 12 h to assess treatment response. In cases where perfusion deficits persisted, second-tier endovascular therapies were considered based on interdisciplinary decision-making.

DCI-related cerebral infarction was defined as new hypodensities on non-contrast CT scans conducted during the patient’s intensive care unit stay, which were not present on prior imaging and not attributable to surgical or endovascular procedures [19, 20]. The timing of infarction was determined as the number of hours from aneurysm rupture to the time at which the infarct was first detected on CT imaging.

### Data Collection and Handling

Invasive arterial blood pressure and intracranial pressure (ICP) were continuously recorded via standard transducers, with mean arterial pressure (MAP) and ICP data sampled at 125 Hz. Brain tissue oxygenation (PtiO₂) was collected at 1 Hz. Data acquisition was performed using the MPR2 logO Datalogger (Raumedic, Germany) until July 2018, after which the Moberg CNS Monitor (Moberg Research, USA) was used. Calibration artifacts and signal disruptions were excluded to ensure data integrity.

Cerebrovascular reactivity was assessed via the pressure reactivity index (PRx), computed in ICM+ [21] software (University of Cambridge, UK) using moving Pearson correlations between 10-second averages of MAP and ICP over a 5-minute sliding window with 80% overlap, and 50% missing data limit [12, 22].

Microdialysis catheters (µdialysis, Sweden) were perfused continuously at 0.3 µL/min with crystalloid solution. Dialysate samples were collected every three hours under standard conditions or hourly during metabolic deterioration. Analytes, including lactate, pyruvate, glutamate, and glycerol, were quantified bedside using the ISCUSflex analyzer. Lactate and pyruvate reflect glycolytic and oxidative metabolism, with their ratio (LPR) indicating the balance between anaerobic and aerobic energy production [23, 24]. Glutamate is an excitatory neurotransmitter whose extracellular accumulation indicates excitotoxicity and impaired astrocyte-neuron recycling [25]. Glycerol is a membrane phospholipid degradation product indicating structural cellular injury [25]. Missingness in microdialysis data, commonly due to clinical procedures such as imaging-related transport, was systematically evaluated. To ensure sufficient data density for modeling, inclusion in multivariate analyses required ≥ 30% of expected hourly microdialysis data (primarily glutamate or glycerol). This threshold aims to balance data quality with cohort size.

The spatial relationship between neuromonitoring probes and infarcted brain regions was assessed using vascular territory maps derived from the extended Alberta Stroke Program Early CT Score (ASPECTS). Probes were considered within or adjacent to the infarct if the affected territory involved the ipsilateral A1, A2, M1, M4, or M5 regions [3, 26].

This study was designed as an exploratory cohort analysis focused on physiological changes surrounding radiological detection of DCI-related infarction. All data were temporally aligned to the hour of infarction diagnosis based on CT imaging. High-frequency parameters (MAP, ICP, PtiO₂) were aggregated into hourly means or medians (based on the underlying distribution) to synchronize with microdialysis sampling. Paired analyses compared pooled 24-hour windows, defined by per-patient medians, centered around identified troughs and peaks in the data models.

### Statistical and Data Analysis

All statistical analyses and visualizations were conducted using a combination of R (v4.4.0; www.r-project.org) within RStudio (v2024.12.0 + 467) and Python (v3.13). Data wrangling and visualization in R were performed using the *tidyverse* suite, and generalized additive models (GAMs) were implemented with the *mgcv* package. In Python, data manipulation was carried out using *pandas* and *numpy*, while machine learning analyses employed *scikit-learn* (v1.3+), and model interpretability was supported by *SHAP* (v0.42+).

Descriptive statistical analyses followed standard best practices. The distribution of continuous variables was assessed using histograms, quantile–quantile plots, and the Shapiro–Wilk test when appropriate. Variables with a normal distribution are presented as mean ± standard deviation (SD) and were compared using unpaired or paired t-tests depending on the study design. Non-normally distributed variables are reported as median and interquartile range (IQR; Q1–Q3) and analyzed using Mann–Whitney U tests or Wilcoxon signed-rank tests for paired data. Categorical variables are presented as counts and percentages and were assessed using the chi-square test or Fisher’s exact test when cell counts were sparse. All statistical tests were two-sided, with a significance threshold set at *p* < 0.05. The study conforms to the STROBE (Strengthening the Reporting of Observational Studies in Epidemiology) guidelines for observational data reporting.

### Generalized Additive Models (GAMs)

To investigate non-linear temporal dynamics in continuous monitoring data, generalized additive models (GAMs) were used and fitted to hourly data. These models made use of thin-plate regression splines for smooth term estimation and included a continuous-time autoregressive term to account for temporal autocorrelation. Smoothing parameters were optimized via restricted maximum likelihood. To identify critical temporal dynamics preceding cerebral infarction, the first derivative of each fitted GAM smooth function was computed. This derivative analysis was used to determine two key time points: (i) the trough, defined as the lowest point in the model-predicted trajectory, presumably prior to infarction detection, and (ii) the peak, defined as the highest predicted value in proximity to the infarction event. These time points were then used to define summary time windows to compare against each other.

For each individual, 24-hour windows centered around the identified troughs and peaks were extracted. Within each window, the median value of the physiological parameter was calculated to account for skewed data distributions. These per-patient median values were then pooled across the cohort, allowing for a paired comparison between trough and peak periods.

### Machine Learning Approach for Multivariate Time Series Analysis

The objective of this machine learning analysis was to characterize the temporal and physiological patterns that distinguish monitoring periods associated with DCI-related infarction development from those in patients who did not develop DCI. We aimed to identify the multimodal biomarker signatures and temporal dynamics that characterize infarction progression following subarachnoid hemorrhage.

Initially, generalized linear mixed-effects models (GLMMs) with spline terms were explored to account for repeated measures and potential non-linear trajectories over time. However, these models failed to converge due to the computational burden of simultaneously modeling four correlated time series across 69 patients over a 73-hour observation window with substantial missing data.

To address these limitations, particularly missing data, complex temporal dependencies, and non-linear relationships, a Random Forest classifier was employed. Random Forests were selected for their robustness to missing values (via surrogate splits and bootstrap sampling), ability to capture non-linear interactions without prior distributional assumptions, compatibility with mixed data types and skewed distributions, and interpretability through feature importance and SHAP (SHapley Additive exPlanations) analysis. Overfitting was minimized using ensemble averaging and built-in cross-validation. Missing data were handled natively by the Random Forest algorithm through surrogate splits. When the primary splitting variable is missing for a given observation, the algorithm identifies an alternative variable that produces a similar partition of the data, allowing the observation to traverse the decision tree without requiring imputation. This approach utilizes all available data while avoiding assumptions associated with imputation methods.

The dataset was aligned to a 73-hour analysis window extending from 48 h before to 24 h after the time of DCI-related infarction detection, as determined by generalized additive model (GAM) results. For the no-DCI control group, monitoring data were time-aligned using the median time point of infarction detection from the DCI-infarction group, ensuring comparable temporal windows across both groups. The task was framed as binary classification, where each patient’s complete monitoring window within this timeframe constituted one observation labeled according to clinical outcome: DCI-related infarction or no DCI. Patients not progressing to infarction served as a control group, though this group exhibited high levels of missing data within the time window of interest.

To capture short-term trends and temporal information without using full temporal sequences, structured feature engineering was applied to the key biomarkers: brain tissue oxygenation (PtiO₂), pressure reactivity index (PRx), glutamate, and glycerol. For each biomarker, the instantaneous hourly value was recorded with rolling means over the preceding six and twelve hours, representing moving averages that summarize recent physiological history, and a six-hour slope calculated as the rate of change over the previous six-hour period, representing short-term temporal dynamics. The 6-hour and 12-hour intervals were selected to capture short- and intermediate-term physiological trends that are clinically meaningful in the context of DCI monitoring, corresponding approximately to standard clinical observation and intervention timeframes in neurocritical care. Alternative window lengths were explored during pilot analysis but yielded similar patterns. This feature engineering approach, encompassing instantaneous values and derived temporal features, reflects typical clinical observation horizons (6–12 h) in neurocritical care practice.

To ensure robust model evaluation and avoid data leakage, the dataset was split at the patient level rather than at the observation level. Patients were randomly assigned to training (80%) or test (20%) sets using stratified sampling to maintain class balance, ensuring that all temporal observations from a given patient appeared exclusively in either the training or test set, never both. This patient-level splitting approach ensures that model performance reflects generalization to entirely new patients rather than merely recognizing patterns from patients already seen during training.

The Random Forest model was configured with 100 trees, balanced class weights to account for the unequal distribution of infarction *versus* no-DCI cases, and regularization parameters (maximum depth of 10, minimum samples per split of 20, minimum samples per leaf of 10) to prevent overfitting. The training set comprised 55 patients (3,965 observations) and the test set 14 patients (1,022 observations), both maintaining approximately 34% infarction prevalence.

Performance was assessed using accuracy, area under the receiver operating characteristic curve (AUC-ROC), sensitivity, and specificity. Feature importance was quantified using mean decrease in impurity from the Random Forest, and SHAP values were computed to provide model-agnostic interpretability, revealing how individual features contribute to predictions and highlighting the physiological patterns most strongly associated with infarction development.

## Results

### Patient Characteristics

**Fig. 1:**
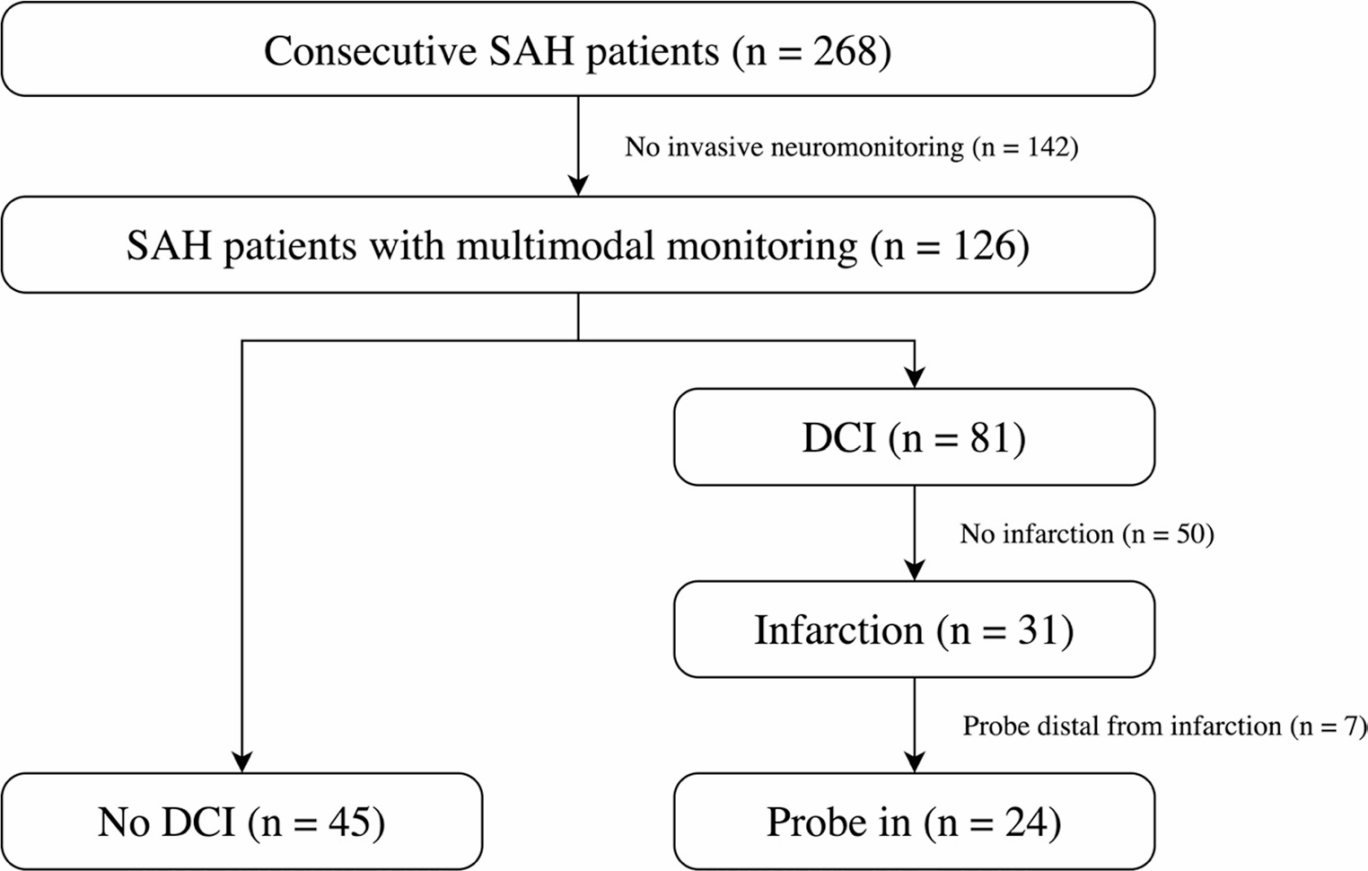
Inclusion flow chart DCI, delayed cerebral ischemia; SAH, aneurysmal subarachnoid hemorrhage. Probe in, refers to probes considered within or adjacent to the infarcted regions as assessed using vascular territory maps derived from the extended Alberta Stroke Program Early CT Score (ASPECTS). Probes are labeled as “in” if the infarction involved the ipsilateral A1, A2, M1, M4, or M5 regions

During the inclusion period (2014–2020), 268 patients with aneurysmal subarachnoid hemorrhage were treated. Due to their initial unconscious state or later loss of consciousness, 126 patients underwent invasive multimodal neuromonitoring, including PtiO₂ and/or cerebral microdialysis. Among these, 81 patients (64.3%) were diagnosed with DCI. Thereof, 31 patients developed DCI-related infarction (infarction group). Probes were placed a median of one day (IQR; 0 to 3) after SAH. The invasive monitoring probe was located within or near the infarcted area in 24 patients. The final analysis included these 24 patients and the 45 patients without the diagnosis of DCI as a control group (no DCI group). A flowchart of patient inclusion is presented in Fig. 1. The full cohort (no DCI + infarction) had a mean age of 58.2 ± 12.1 years, and 50 patients (72.5%) were female. Patient demographics, as well as hemorrhage and treatment characteristics, are detailed in Table 1. Cerebral infarction caused by DCI was detected after a median of 7.5 (5.8–11.0) days. Repeated measures data from the no DCI group were synchronized in time based on 12:00 am of the eighth post-ictus day.

**Table 1:** Demographic and hemorrhage-specific characteristics of included subarachnoid hemorrhage patients stratified by incidence of DCI or DCI-related infarction

	All	No DCI	Infarction	univariate *p*-value
	*n* = 69	*n* = 45	*n* = 24	
Demographics				
age - yrs. - mean ± SD (range)	58.2 ± 12.1	61.2 ± 11.2	52.6 ± 12.0	**0.006**
sex - female / male - no. (%)	50 (72.5) / 19 (27.5)	31 (68.9) / 14 (31.1)	19 (79.2) / 5 (20.8)	0.530
Comorbidities				
Arterial hypertension	30 (43.5)	21 (46.7)	9 (37.5)	0.578
BMI	25.5 ¬± 4.8	25.9 ± 4.2	25.0 ± 5.9	0.562
Smoking	23 (33.3)	15 (33.3)	8 (33.3)	1
Type II diabetes	3 (4.3)	2 (4.4)	1 (4.2)	1
Aneurysm location - no. (%)				
anterior circulation	58 (84.1)	37 (82.2)	21 (87.5)	0.822
diameter - mean ± SD	6.3 (4.7–11.0)	7.0 (5.0–11.0)	5.4 (4.0–8.8)	0.160
clipping / endovascular	29 (42.6) / 39 (57.4)	19 (42.2) / 26 (57.8)	10 (41.7) / 13 (54.2)	1
Hemorrhage severity				
WFNS grade - no. (%)				0.515
grade 1	15 (21.7)	11 (24.4)	4 (16.7)	
grade 2	8 (11.6)	7 (15.6)	1 (4.2)	
grade 3	13 (18.8)	8 (17.8)	5 (20.8)	
grade 4	20 (29.0)	11 (24.4)	9 (37.5)	
grade 5	13 (18.8)	8 (17.8)	5 (20.8)	
poor-grade SAH (WFNS 3–5)	45 (65.2)	26 (57.8)	19 (79.2)	
modified Fisher scale - no. (%)				0.741
grade 1	11 (15.9)	8 (17.8)	3 (12.5)	
grade 2	9 (13.0)	7 (15.6)	2 (8.3)	
grade 3	21 (30.4)	13 (28.9)	8 (33.3)	
grade 4	28 (40.6)	17 (37.8)	11 (45.8)	
ICH	28 (40.6)	19 (42.2)	9 (37.5)	0.902
Infarction				
Lag post ictus (days) - mean ± SD			7.5 (5.8–11.0)	
Total volume (mL)			57.7 (45.4–131.6)	

*Acomm *anterior communicating artery, *BMI* body mass index, *DCI* delayed cerebral ischemia, *mL* milliliter *SAH *aneurysmal subarachnoid hemorrhage, *SD *standard deviations, *WFNS *World Federation of Neurosurgical Societies grading Significant p-values (< 0.05) are written in bold

### Temporal Trends Around Infarct Detection

The GAM analysis revealed distinct temporal patterns in physiological parameters between patients with and without cerebral infarction.

**Fig. 2:**
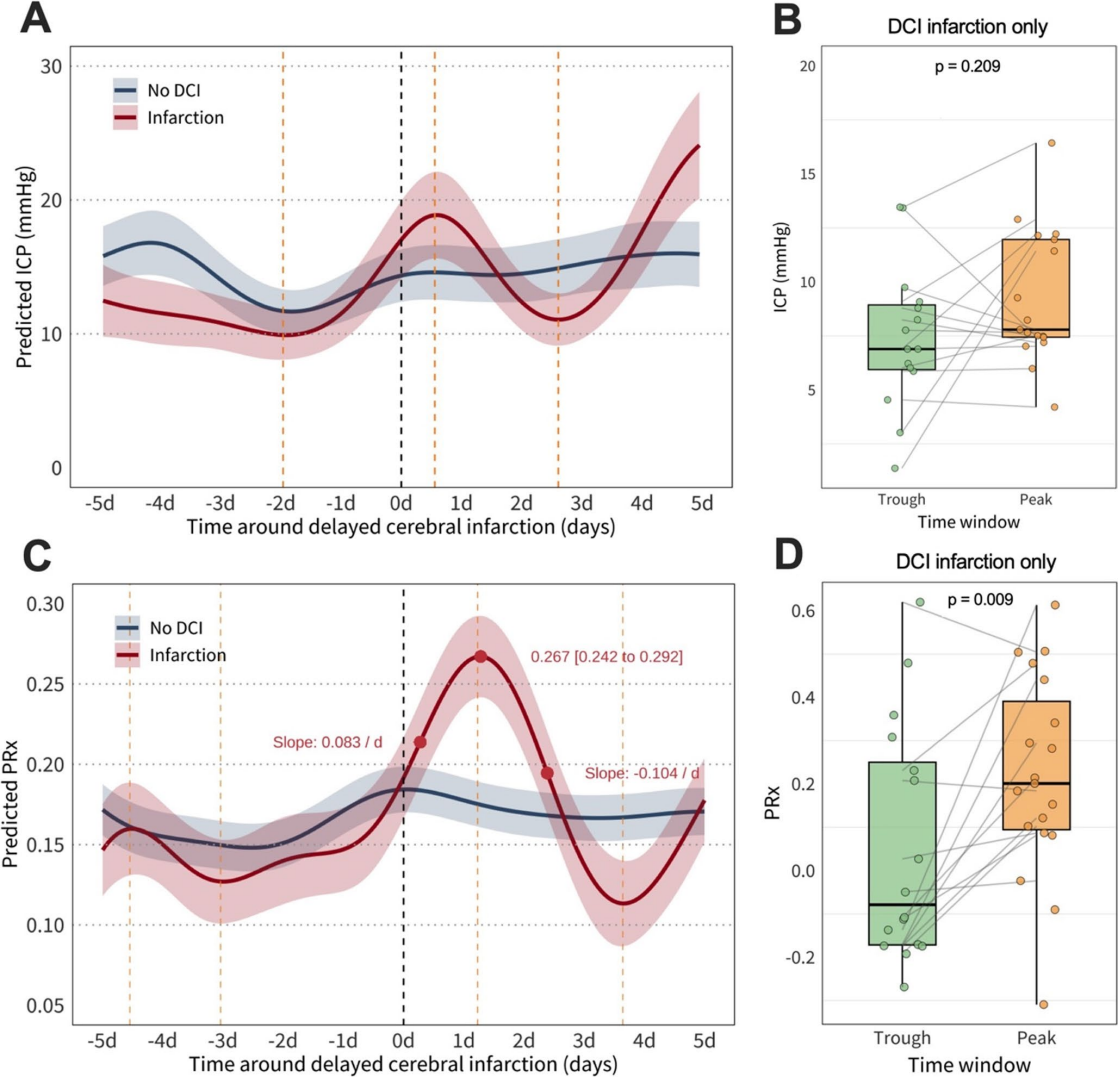
GAM predicted ICP and PRx over 10 days around the detection of DCI-related infarction (**A**-**C**) and paired comparison of pooled trough and peak values of ICP (B) and PRx (**D**). **A **& **C **represent a Generalized Additive Model (GAM) predicted time courses with 95% confidence intervals (ribbon). The black dashed line denotes the time point of detection of DCI-infarction. The yellow dashed lines denote troughs and peaks in the GAM prediction of the infarction group. In C, the maximum rise and fall as well as peak values with 95% confidence intervals, are indicated. **B **& **D**. Paired comparison of patients’ values coming from the 24 hours surrounding the identified trough versus 24 hours surrounding the identified peak, with Wilcoxon signed-rank test results. d, days around the detection of DCI-related infarction; DCI, delayed cerebral ischemia; ICP, intracranial pressure; mmHg, millimeters of mercury; PRx, pressure reactivity index.

Intracranial pressure (ICP) dynamics (Fig. 2A–B) differed notably between groups. In the no DCI group, ICP remained stable between 15 and 17 mmHg. The infarction group displayed a biphasic trajectory: a trough one to two days before infarction detection, followed by a sustained rise peaking around day + 1 after detection of infarction, with predicted values nearing 25 mmHg. In patient with infarction, median ICP increased from 6.898 mmHg (IQR: 6.035 to 9.078) during the trough to 8.766 mmHg (IQR: 7.424 to 12.089) during the peak, though this difference was not statistically significant (Wilcoxon signed-rank test, *p* = 0.209).

Pressure reactivity index (PRx) patterns (Fig. 2C–D) were more pronounced in the infarction group. PRx declined to a trough approximately three days prior to infarct detection, then rose sharply at 0.083 per day, peaking between day + 1 and + 2 at 0.267 (95% CI: 0.242 to 0.292), followed by a decline of − 0.104 per day. In the no DCI group, PRx remained stable at around 0.170. Comparison of 24-hour windows around the trough and peak of the infarction group, showed a median PRx increase of 0.293 between trough (-0.078; IQR: − 0.181 to 0.249) and peak (0.215; IQR: 0.096 to 0.385) in the infarction group (*p* = 0.009).

**Fig. 3:**
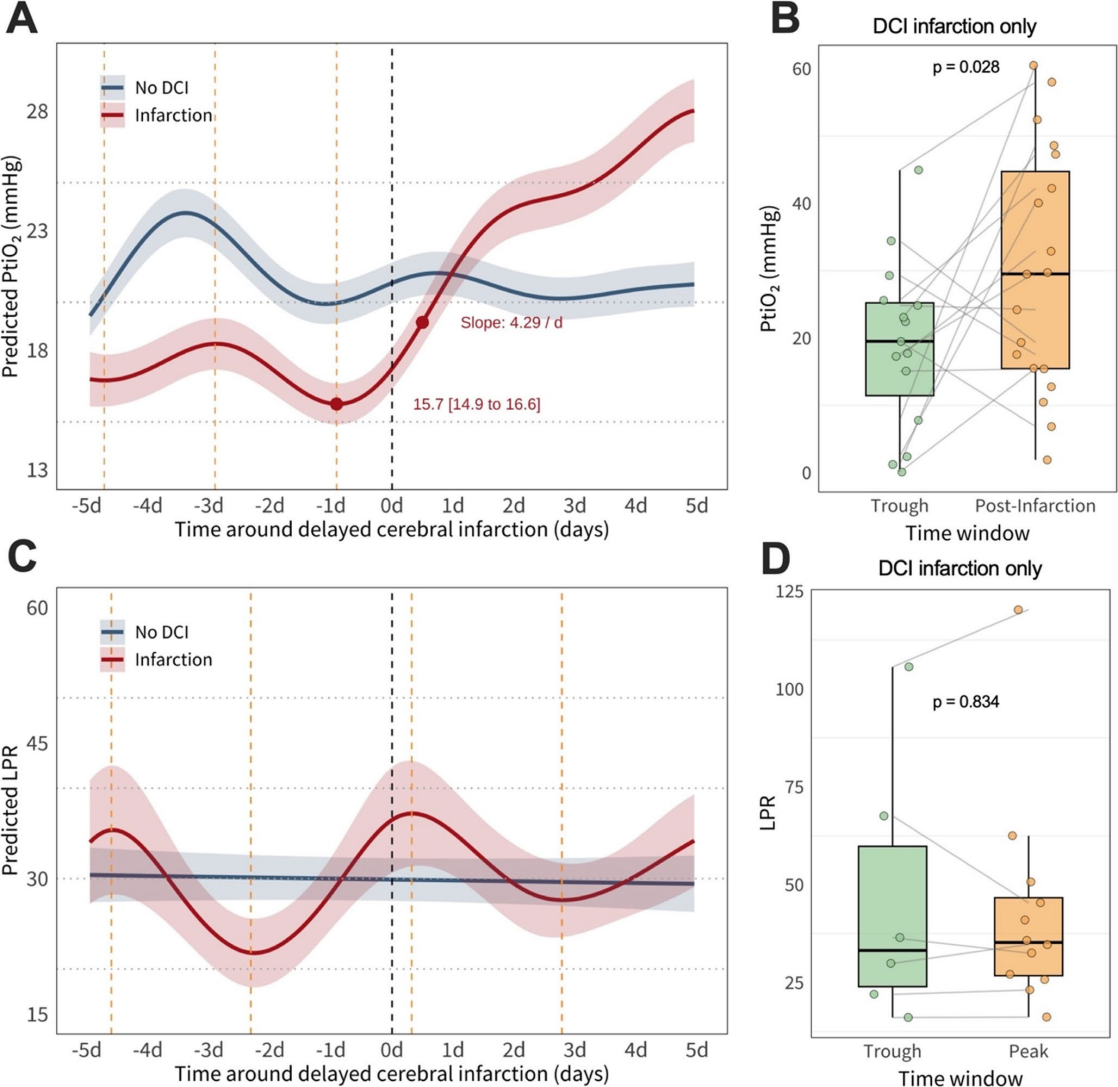
GAM predicted brain tissue oxygen tension PtiO2 and lactate-to-pyruvate ratio (LPR) over 10 days around the detection of DCI-related infarction (**A**-**C**) and paired comparison of pooled trough and peak values of PtiO2 (**B**) and LPR (**D**). **A **& **C **represent a Generalized Additive Model (GAM) predicted time courses with 95% confidence intervals (ribbon). The black dashed line denotes the time point of detection of DCI-infarction. The yellow dashed lines denote troughs and peaks in the GAM prediction of the infarction group. In A, the maximum rise and as well as trough values with 95% confidence intervals, are indicated. **B **& **D**. Paired comparison of DCI-infarction patients’ values coming from the 24 hours surrounding the identified trough versus the hour of the peak, with Wilcoxon signed-rank test results. As described in the results, for PtiO2, no post-infarction peak was identified, and therefore the 5-day window after infarct detection was used for paired analysis, hence the labeling in panel B. Post-Infarction and not Peak. d, days around the detection of DCI-related infarction; DCI, delayed cerebral ischemia; LPR, lactate-to-pyruvate ratio; mmHg, millimeters of mercury; PRx, pressure reactivity index; PtiO2, brain tissue oxygen tension

Brain tissue oxygenation (PtiO₂) also showed group differences (Fig. 3A–B). In the pre-detection phase, the infarction group had a lower baseline PtiO₂ (~ 17 mmHg) than the no DCI group (~ 20 mmHg). One day prior to infarction detection, PtiO₂ dropped to 15.7 mmHg (95% CI: 14.9 to 16.6). This was followed by an increase in PtiO_2_ with a maximal slope of 4.29 mmHg/day, shortly after infarct detection. The no DCI group maintained stable PtiO₂ near 21 mmHg. In the infarction group, no clear peak was identified, and instead, a pooled 5-day post-infarction window was used for comparison. Median PtiO₂ increased significantly from 19.5 mmHg (IQR: 11.4 to 25.2, *n* = 15) during the trough to 29.5 mmHg (IQR: 15.5 to 44.7, *n* = 19; *p* = 0.028), possibly reflecting therapeutic interventions or autoregulatory compensation.

Lactate-to-pyruvate ratio (LPR) exhibited no consistent temporal trend (Fig. 3C–D). The infarction group showed fluctuating peaks and troughs, while the no DCI group remained stable around 30. Median LPR values rose slightly from 33.2 (IQR: 24.0 to 59.8, *n* = 6) to 35.3 (IQR: 26.8 to 46.7, *n* = 12) in the infarction group, with no significant difference (*p* = 0.834).

**Fig. 4:**
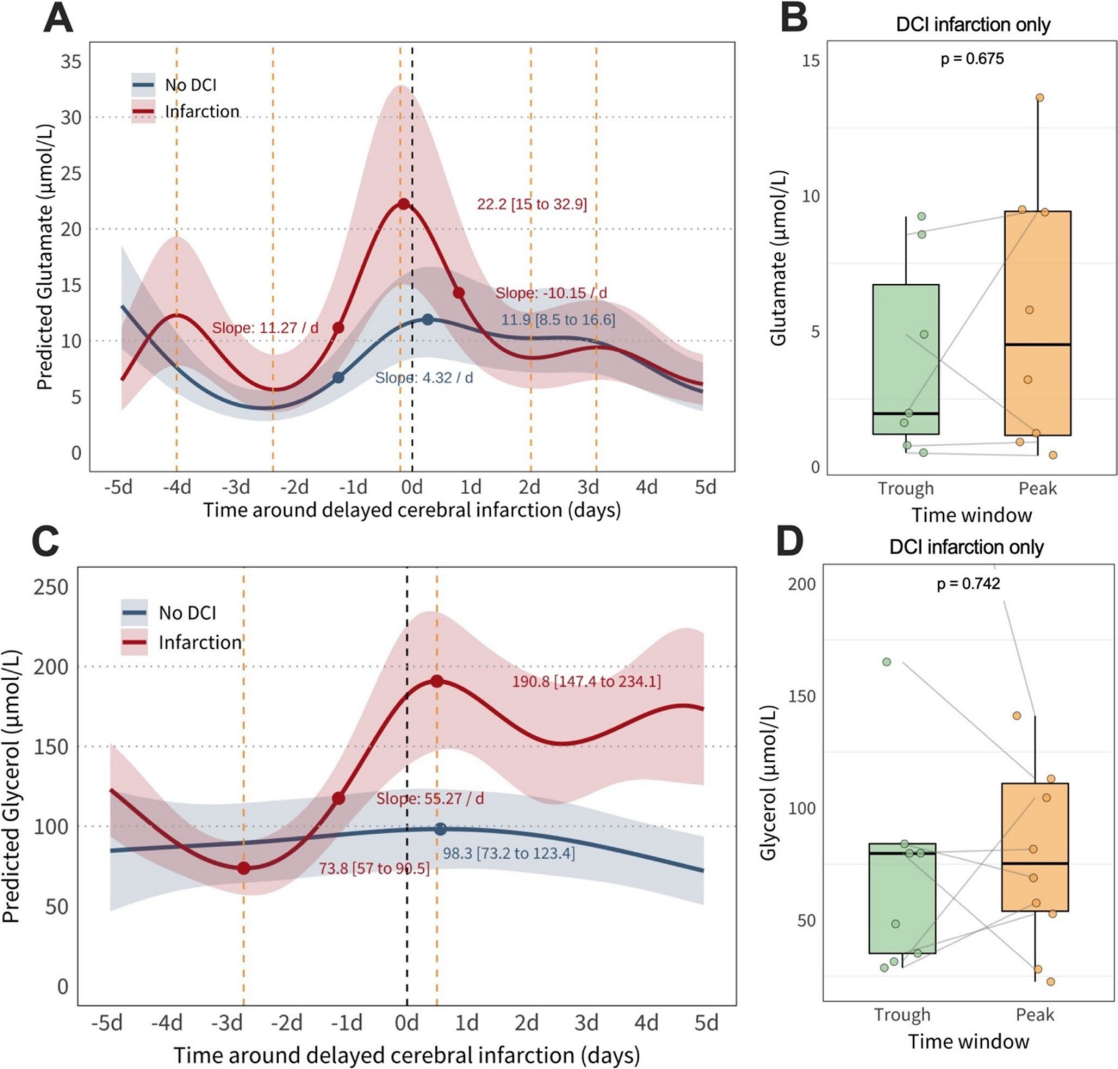
GAM predicted levels of Glutamate (µmol/L) and Glycerol (µmol/L) over 10 days around the detection of DCI-related infarction (**A**-**C**) and paired comparison of pooled trough and peak values of Glutamate (**B**) and Glycerol (**D**) levels. **A **& **C **represent a Generalized Additive Model (GAM) predicted time courses with 95% confidence intervals (ribbon). The black dashed line denotes the time point of detection of DCI-infarction. The yellow dashed lines denote troughs and peaks in the GAM prediction of the infarction group. In **A **& **C**, the maximum slopes as well as trough and peak values with 95% confidence intervals are indicated. **B **& **D**. Paired comparison of DCI-infarction patients coming from the 24 hours surrounding the identified trough versus the hour of the peak, with Wilcoxon signed-rank test results. d, days around the detection of DCI-related infarction; DCI, delayed cerebral ischemia; L, liter; µmol, micromole

Glutamate dynamics (Fig. 4A–B) showed a pronounced increase in the infarction group, consistent with excitotoxic activity. Approximately two days before detection, glutamate rose at 11.270 µmol/L/day, peaking at 22.2 µmol/L (95% CI: 15.0 to 32.9) around day 0 to + 1, then declined at − 10.150 µmol/L/day, up to day + 2. In contrast, the no DCI group maintained stable levels between 10.0 and 12.0 µmol/L. Median glutamate rose from 1.960 µmol/L (IQR: 0.880 to 9.400, *n* = 9) to 4.505 µmol/L (IQR: 1.163 to 8.328, *n* = 8), though this was not statistically significant (*p* = 0.675).

Glycerol levels (Fig. 4C–D) displayed a different time trend. In the infarction group, levels increased with a maximum slope of 55.3 µmol/L/day, peaking at 190.8 µmol/L (95% CI: 147.4 to 234.1) around day + 1 and remained elevated through day + 5. The no DCI group peaked at 98.3 µmol/L (95% CI: 73.2 to 123.4) with a gradual decline. In the infarction group, the median was 79.8 µmol/L (IQR: 35.2 to 84.1) at the trough and 75.3 µmol/L (IQR: 54.0 to 111.0) at the peak (*p* = 0.742). The lower median at peak, despite higher predicted values, likely reflects skewed data with outliers.

### Analysis of Multimodal Neuromonitoring Patterns

The Random Forest model demonstrated good discriminative performance in characterizing DCI-related infarction, achieving an area under the curve (AUC) of 0.814 on the test set, with 75.9% overall accuracy, 68.9% sensitivity, and 79.8% specificity.

Feature importance analysis revealed the hierarchy of biomarker patterns distinguishing the analysis window associated with infarction development from non-infarction monitoring periods (corresponding to the time around DCI detection or the time-aligned control period for the no-DCI group). Glutamate temporal patterns dominated, with the 12-hour rolling average, computed using the preceding 12 h of data, contributing 16.4% of total feature importance, followed by PtiO₂ temporal patterns (12-hour mean 15.2%, 6-hour mean 12.4%) and the instantaneous PtiO₂ value at the current hour (8.1%), as well as the PRx 12-hour average (9.2%). Notably, temporal averaging features, which summarize the immediately preceding 6–12 h, consistently outperformed instantaneous measurements, indicating that sustained biomarker trends over these horizons were more characteristic of infarction development than momentary values. Feature importances are aggregated over all patient-hours in the analysis window.

SHAP analysis provided more insights into how specific biomarker values influenced classification. High glutamate 12-hour averages (visualized as red dots in the figure) consistently contributed positive SHAP values toward infarction classification, while low glutamate levels (blue dots) contributed negative values toward no-DCI classification. Similarly, higher PtiO₂ values and sustained elevations showed positive associations with infarction detection, consistent with our previous findings of concerning PtiO₂ patterns. PRx demonstrated that sustained impairment (higher 12-hour averages) was associated with infarction development, while glycerol elevation patterns, though less prominent, contributed to the multivariate characterization.

**Fig. 5:**
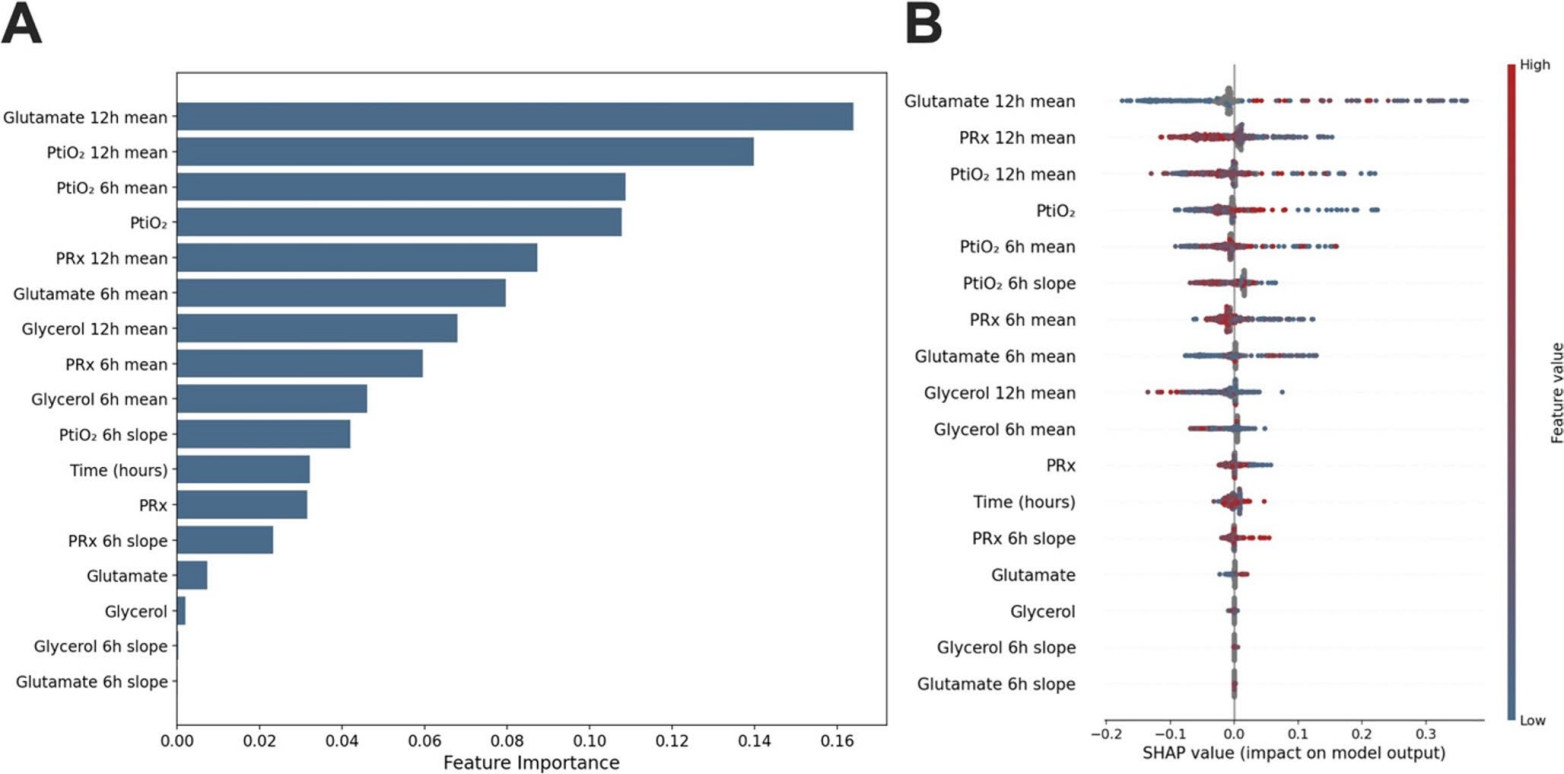
Random Forest feature importance and SHAP value analysis for multimodal neuromonitoring prediction of infarction detection.(**A**) Feature importance ranking from the Random Forest model showing the relative contribution of each biomarker feature to infarction prediction. Glutamate 12-hour rolling mean, calculated as a moving average over the preceding 12 hours, was the strongest predictor (16.4 % importance), followed by PtiO₂ temporal patterns (12-hour and 6-hour rolling means). Temporal averaging features consistently outperformed instantaneous measurements, indicating that sustained biomarker changes over 6–12-hour periods were more predictive than momentary values.(**B**) SHAP (SHapley Additive exPlanations) summary plot illustrating the impact of individual feature values in infarction classification. Each dot represents a patient-hour sample; position on the x-axis indicates SHAP value magnitude (positive values increase infarction probability, negative values decrease it). Color intensity represents feature value (blue = low, red = high). High glutamate 12-hour averages (red dots) consistently contributed positive SHAP values toward infarction prediction, while elevated PtiO₂ and impaired PRx patterns showed similar associations. These findings indicate that higher and sustained biomarker elevations increased infarction probability.Model performance: AUC = 0.884, accuracy = 78.3 %, sensitivity = 68.9 %, specificity = 83.1 % on held-out test data.

The model identified (Fig. 5) that multimodal pattern recognition characterized infarction development better than individual biomarker thresholds, with all four monitoring modalities (PRx, PtiO₂, glutamate, and glycerol) contributing to the discriminative features distinguishing the infarction-associated monitoring period from control periods.

## Discussion

The objective of this study was to characterize the simultaneous, temporally resolved evolution of multimodal neuromonitoring parameters during the transition from delayed cerebral ischemia to irreversible infarction in patients with aneurysmal subarachnoid hemorrhage. While individual biomarkers of ischemic injury are well-established, the integrated temporal dynamics of these parameters during the critical transition to infarction, preserved at native sampling resolution and aligned to radiological detection, have not been systematically described, limiting their bedside interpretability and clinical utility for guiding therapy escalation or de-escalation decisions. In this study, we identified distinct physiological trajectories associated with the development of delayed cerebral infarction in patients with aneurysmal subarachnoid hemorrhage. Patients who developed infarction exhibited characteristic changes across multiple neuromonitoring parameters, including deterioration in cerebrovascular reactivity and alterations in brain tissue oxygenation. Notably, a transient surge in glutamate levels preceded infarction, aligning with mechanisms of excitotoxic injury, while glycerol concentrations showed a sustained elevation, suggestive of ongoing membrane breakdown. Among these parameters, impaired pressure reactivity also appeared closely linked to infarction risk. While microdialysis markers alone were limited in their discriminatory ability, integrating them into a multimodal framework might help to stratify patients by risk, where specific physiological profiles could be associated with higher probabilities of DCI-related infarction.

While the pathophysiological relationships between glutamate elevation, glycerol release, and cerebrovascular reactivity impairment have been well established in prior work, our study extends this knowledge in clinically meaningful ways. Previous investigations typically relied on substantial temporal data aggregation, which obscures the dynamic evolution of metabolic derangement and limits bedside interpretability in a timeline disease such as SAH. Our approach preserved the native sampling frequency of cerebral microdialysis, aligned all physiological parameters to the radiological detection of infarction, and characterized temporal trajectories rather than static thresholds. Building on our previous characterization of invasive neuromonitoring patterns during DCI [11] and treatment responses [27], this work specifically addresses the transition from potentially salvageable tissue to irreversible injury. This distinction carries clinical importance: radiological detection of infarction often lags behind the true onset of irreversible injury due to the low temporal resolution of routine imaging, resulting in continuation of therapies, such as induced hypertension or endovascular spasmolysis, after the therapeutic window has closed. Such futile continuation carries meaningful risks making a bad situation worse, including hemorrhagic transformation of established infarcts, systemic complications from sustained vasopressor therapy, and hazards associated with repeated patient transport for imaging. Bedside recognition of multimodal patterns indicative of completed infarction could facilitate earlier de-escalation of aggressive therapies, trigger timely confirmatory imaging when clinically appropriate, or in some cases obviate unnecessary imaging altogether, thereby reducing both futile interventions and transport-related complications in critically ill patients.

### Brain Tissue Oxygen Delivery

Adequate cerebral oxygenation depends on three steps: convective delivery, diffusion, and cellular utilization [28]. Convective delivery reflects cerebral blood flow and arterial oxygen content, which is determined by hemoglobin concentration, oxygen saturation, and arterial oxygen tension. After oxygen reaches the capillary bed, diffusion into tissue follows the capillary–tissue gradient and is influenced by microvascular integrity and edema. Utilization then depends on mitochondrial function. Disturbance at any step can produce tissue hypoxia.

In the TITAN study of 232 SAH patients, longer time spent with low brain tissue oxygen (PtiO₂ < 20 mmHg), particularly when combined with elevated LPR, was independently associated with worse 6-month outcome and higher mortality [17]. These findings support PtiO₂ as a summary marker of the balance among perfusion, diffusion, and metabolism.

In our cohort, PtiO₂ fell before infarct detection and then rose afterward. This might be explained by the self-fulfilling prophecy that imaging is triggered by neuromonitoring, in this case persistent low oxygenation levels. The post-infarct increase likely reflects reduced local extraction in necrotic tissue, together with therapy that augments convective delivery, such as induced hypertension or ventilatory adjustments. PtiO₂.

Intraparenchymal PtiO₂ monitoring provides continuous regional data but has limitations. The signal can be influenced by dissolved oxygen within the microvasculature, and the probe location relative to the core and penumbra affects interpretability. Normobaric hyperoxia, for example, can increase PtiO₂ despite reduced cerebral blood flow. Thus, PtiO₂ like other physiological signals, is dynamic and should be interpreted in its temporal and therapeutic context rather than as a fixed threshold. It might be better perceived as a contextual indicator of regional oxygen balance, not a direct measure of tissue viability.

### Previous Microdialysis Research in SAH

Early clinical cerebral microdialysis studies in SAH established the feasibility of bedside metabolic monitoring and linked disturbances in extracellular glucose, lactate, lactate-to-pyruvate ratio, glutamate, and glycerol to secondary ischemic injury [8, 9, 29]. However, much of the foundational literature from early 2000s was conducted within a diagnostic paradigm that largely equated secondary injury with angiographic vasospasm and/or transcranial Doppler velocity increases as the principal endpoint, rather than contemporary DCI definitions. Methodologically, many studies relied on substantial post hoc data reduction (e.g., daily medians, pooled 24-hour summaries, or baseline windows) and comparisons across broad clinical categories or outcomes, which is informative for associations with disease severity but limits bedside interpretability of evolving trajectories in a timeline disease such as SAH. Even when CMD was correlated with perfusion imaging (e.g., PET-derived regional cerebral blood flow), these correlations were necessarily tied to isolated imaging “snapshots” and phase-based aggregation (e.g., multi-day medians), rather than event-locked time-series characterization of the transition from DCI to DCI-related infarction [29]. In addition, external validity of earlier series is constrained by major shifts in contemporary care: a far greater proportion of aneurysms are now treated endovascularly, DCI concepts have broadened beyond large-vessel vasospasm, and DCI therapy has evolved from “triple-H” regimens toward euvolemic induced hypertension with increasing use of endovascular rescue strategies. Against this background, our analysis intentionally preserves temporal information at the sampling resolution of cerebral microdialysis, by aligning multimodal monitoring to infarct detection and reducing higher-frequency signals to hourly summaries (rather than coarser daily aggregation), enabling assessment of sustained trends and slopes that may better reflect clinically relevant transition dynamics. A comprehensive synthesis of CMD applications, interpretive pitfalls, and the emphasis on trend-based interpretation is provided in the state-of-the-art review by Helbok and colleagues, which also highlights the need for larger, prospective, intervention-linked studies in SAH [23].

### Microdialysis in Ischemic Stroke

While DCI-related infarction and thromboembolic stroke involve different pathophysiology, their final common pathway of cerebral cell death, overlaps. Although microdialysis studies in human ischemic stroke are limited, they provide valuable insights into metabolic disturbances in the infarcted brain. Berger et al. investigated the metabolic effects of moderate hypothermia in patients with malignant middle cerebral artery infarctions using microdialysis [30]. Their data revealed distinct metabolic signatures across infarcted, penumbral, and non-infarcted tissue with excessive levels of glutamate (453 µmol/L) and glycerol (1187 µmol/L) indicative of irreversibly tissue damage.

### Glutamate-Mediated Excitotoxicity in Cerebral Ischemia

Excitotoxicity is a well-established cellular mechanism in cerebral ischemia, driven by disrupted metabolic homeostasis and excessive release of neurotransmitters such as glutamate. These metabolites bind to NMDA receptors, leading to calcium influx and further disruption of neuronal homeostasis. Lack of substrate delivery disables the energy-dependent cycling of astrocyte-neuron glutamate/glutamine, further resulting in the increase of extracellular glutamate levels, continuing the vicious cycle [7, 31].

Recent work by Wang et al. has extended this paradigm by demonstrating that glutamate also acts as a positive allosteric modulator of acid-sensing ion channels (ASIC1a), exacerbating neuronal injury under ischemic conditions [32]. Their study identified a novel glutamate-binding site on ASIC1a, which increases the channel’s sensitivity to mild acidosis and amplifies calcium-mediated toxicity in both in vitro and in vivo stroke models. This pathway operates independently of NMDA receptors, suggesting that ASIC1a contributes to early ischemic injury and may represent a more selective therapeutic target.

Our findings, showing a transient glutamate surge preceding infarction, support the relevance of these mechanisms in the setting of DCI and its progression to infarction. Despite the inherent uncertainty in infarct timing due to reliance on routine imaging, the temporal relationship between glutamate elevation and infarct detection highlights its potential role as an upstream mediator of injury, rather than a mere byproduct of necrosis.

### Metabolic Failure

The variable behavior of traditional metabolic markers, such as the lactate-to-pyruvate ratio, in the setting of DCI raises questions about the underlying mechanisms. Classic models of ischemia emphasize energy failure as the primary insult. However, work by Yatsu et al. using a rabbit model of global ischemia demonstrated that mitochondrial adenosine triphosphate synthesis can be preserved for several minutes following ischemic onset, with rapid recovery upon reperfusion [33]. These findings suggest that ischemic vulnerability may instead stem from failure of energy-dependent processes, such as ion transport and synaptic function, rather than metabolic collapse per se. This distinction may explain the observed inconsistent changes in e.g., the lactate-to-pyruvate ratio observed in this study.

### Glycerol as a Marker of Membrane Breakdown

In this study, glycerol levels rose prior to infarct detection and plateaued thereafter, consistent with its role as a marker of membrane phospholipid degradation. This pattern mirrors findings by Hillered et al., who reported marked increases in interstitial glycerol during episodes of secondary ischemia in SAH patients [34]. These increases were independent of systemic glycerol levels, indicating a cerebral origin. The accumulation of glycerol correlated with elevations in glutamate, suggesting a common cascade of excitotoxic injury and membrane breakdown.

### Implications for Clinical Management

Given the exploratory nature of this study, definitive practice recommendations would be premature. However, our findings suggest potential applications of the multimodal signatures which was identified. Particularly the combination of impaired cerebrovascular reactivity, sustained glutamate elevation, and progressive glycerol rise, may inform bedside risk stratification. The demonstration that 6–12-hour windows outperform instantaneous measurements provides support for a potentially still clinically actionable time frame. Or put in other words, a selection of infarctions might theoretically still be successfully prevented however it is unclear how.

#### Limitations

This study was designed to explore temporal patterns and associations between neuromonitoring parameters and DCI-related infarction following aneurysmal subarachnoid hemorrhage. Several important limitations should be acknowledged.

First, the retrospective nature introduces temporal imprecision that limits interpretability. The true timing of infarction onset remains unknown, as imaging is initiated based on variable clinical triggers. The analysis window was anchored to radiologically confirmed infarction, which represents detection rather than true onset. Consequently, physiological changes observed in the “pre-infarction” window may have occurred after actual infarction development, complicating causal inference. In addition, we lack systematic long-term imaging follow-up to definitively confirm that all hypodense regions represented irreversible infarction rather than potentially reversible ischemia or edema, though their evolution from CT perfusion-documented hypoperfusion supports our infarction classification. Also, our binary classification of infarction status does not account for infarction size or the specific vascular territories affected, treating small focal infarctions and large hemispheric infarctions equivalently despite their substantially different clinical implications.

Second, this is a descriptive characterization study rather than a predictive model. We used retrospectively available data to define analysis windows and time-align patients’ monitoring data. The inclusion of data from both before and after the detection time point was intentional to capture the complete physiological signature associated with infarction development but precludes using this model for real-time prediction. The goal is hypothesis-generating pattern characterization rather than prospective clinical decision support.

Third, the comparison groups present inherent limitations. Clinically, the most informative comparison would have been between a DCI-infarction group and a group where DCI was reversed without progression to infarction. However, while patients meeting these criteria existed in our cohort, insufficient high-quality monitoring data in this subset precluded meaningful analysis.

Fourth, class imbalance and modest sample size constrained model development. The infarction group was substantially smaller than the no-DCI control group, limiting statistical power and generalizability despite a large number of hourly observations. The patient-level data split, while methodologically appropriate to avoid data leakage, resulted in a small test set that yields limited precision in performance estimates.

Fifth, feature importance estimates may be affected by multicollinearity among temporal features. The engineered features (rolling averages, slopes) are inherently correlated with instantaneous values and each other, which can dilute individual importance scores and complicate interpretation of independent causal relationships.

Sixth, this single-center analysis may have limited external validity. Generalizability to centers with different monitoring approaches, patient populations, or treatment protocols remains to be established through external validation.

Finally, this study was not designed to evaluate the efficacy of specific treatment interventions targeting neuromonitoring parameters. Whether PtiO₂-directed protocols or other targeted therapies might alter the physiological trajectories described here, and thereby modify the transition from DCI to infarction, remains an important open question warranting prospective investigation.

## Conclusion

This study provides insights into the physiological processes preceding delayed cerebral infarction in patients with aneurysmal subarachnoid hemorrhage. The integration of multimodal neuromonitoring shows distinct trajectories of excitotoxicity, membrane degradation, and autoregulatory failure. A transient glutamate surge and sustained glycerol elevation were observed in association with infarction. While individual microdialysis markers showed variable discriminatory capacity, machine learning analysis confirmed that their combined evaluation with PtiO₂ and PRx substantially enhances predictive performance through synergistic biomarker interactions. The temporal integration of these four monitoring modalities captured complementary aspects. Herein, sustained patterns over multiple hours proved more informative than instantaneous measurements. These exploratory findings highlight the potential of continuous multimodal physiological monitoring for the identification of possible critical transition points in DCI progression. However, the predictive utility remains constrained by data limitations, emphasizing the need for larger, high-resolution datasets to validate these observations and improve risk stratification strategies. 

## Data Availability

The data used in this analysis are available from the authors upon reasonable request for use by qualified researchers.

## Abbreviations

ASIC1a: Acid-Sensing Ion Channels
ASPECTS: Alberta Stroke Program Early CT Score
AUC: rea Under the Curve
CI: Confidence Interval
CT: Computed Tomography
CTP: Computed Tomography Perfusion Scanning
DCI: Delayed Cerebral Ischemia
EVD: External Ventricular Drain
GAM: Generalized Additive Model
GLMM: Generalized Linear Mixed Effects Model
HR: Hazard Ratio
ICP: Intracranial Pressure
IQR: Interquartile Range
L: Litter
LPR: Lactate-to-Pyruvate Ratio
mFisher: Modified Fisher Grading Scale
ml: Milliliter
µmol: Micromole
mmHg: Millimeters of Mercury
mRS: Modified Rankin Scale
PtiO2: Partial Pressure of Brain Tissue Oxygen Expressed in Millimeters of Mercury
PRx: Pressure Reactivity Index
Q1: First Quartile
Q1: Third Quartile
ROC: Receiver Operating Characteristic Curve
SAH: Aneurysmal Subarachnoid Hemorrhage
SHAP: SHapley Additive exPlanations
